# Lipophilic Prodrugs of FR900098 Are Antimicrobial against *Francisella novicida In Vivo* and *In Vitro* and Show GlpT Independent Efficacy

**DOI:** 10.1371/journal.pone.0038167

**Published:** 2012-10-15

**Authors:** Elizabeth S. McKenney, Michelle Sargent, Hameed Khan, Eugene Uh, Emily R. Jackson, Géraldine San Jose, Robin D. Couch, Cynthia S. Dowd, Monique L. van Hoek

**Affiliations:** 1 School of Systems Biology, George Mason University, Manassas, Virginia, United States of America; 2 Department of Chemistry and Biochemistry, George Mason University, Manassas, Virginia, United States of America; 3 Department of Chemistry, George Washington University, Washington, D.C., United States of America; 4 National Center for Biodefense and Infectious Diseases, George Mason University, Manassas, Virginia, United States of America; George Mason University, United States of America

## Abstract

Bacteria, plants, and algae produce isoprenoids through the methylerythritol phosphate (MEP) pathway, an attractive pathway for antimicrobial drug development as it is present in prokaryotes and some lower eukaryotes but absent from human cells. The first committed step of the MEP pathway is catalyzed by 1-deoxy-D-xylulose 5-phosphate reductoisomerase (DXR/MEP synthase). MEP pathway genes have been identified in many biothreat agents, including *Francisella*, *Brucella*, *Bacillus*, *Burkholderia*, and *Yersinia*. The importance of the MEP pathway to *Francisella* is demonstrated by the fact that MEP pathway mutations are lethal. We have previously established that fosmidomycin inhibits purified MEP synthase (DXR) from *F. tularensis* LVS. FR900098, the acetyl derivative of fosmidomycin, was found to inhibit the activity of purified DXR from *F. tularensis* LVS (IC_50_ = 230 nM). Fosmidomycin and FR900098 are effective against purified DXR from *Mycobacterium tuberculosis* as well, but have no effect on whole cells because the compounds are too polar to penetrate the thick cell wall. Fosmidomycin requires the GlpT transporter to enter cells, and this is absent in some pathogens, including *M. tuberculosis*. In this study, we have identified the GlpT homologs in *F. novicida* and tested transposon insertion mutants of *glpT*. We showed that FR900098 also requires GlpT for full activity against *F. novicida*. Thus, we synthesized several FR900098 prodrugs that have lipophilic groups to facilitate their passage through the bacterial cell wall and bypass the requirement for the GlpT transporter. One compound, that we termed “compound 1,” was found to have GlpT-independent antimicrobial activity. We tested the ability of this best performing prodrug to inhibit *F. novicida* intracellular infection of eukaryotic cell lines and the caterpillar *Galleria mellonella* as an *in vivo* infection model. As a lipophilic GlpT-independent DXR inhibitor, compound 1 has the potential to be a broad-spectrum antibiotic, and should be effective against most MEP-dependent organisms.

## Introduction

### 1.1 Methylerythritol phosphate pathway

Isoprenoids are involved in many critical cellular functions. They participate in electron transport, signal transduction, and maintenance of cell wall and membrane structural integrity. All isoprenoids are formed through either the mevalonic acid (MVA) or the methylerythritol phosphate (MEP) pathways [1]. Plants, algae, and bacteria utilize the MEP pathway to generate isopentenyl pyrophosphate (IPP) and dimethylallyl pyrophosphate (DMAPP) from pyruvate and glyceraldehyde-3-phosphate [2]. The MVA pathway is the only pathway used by animals, making enzymes of the MEP pathway attractive targets for novel therapeutics [1].

**Figure 1:**
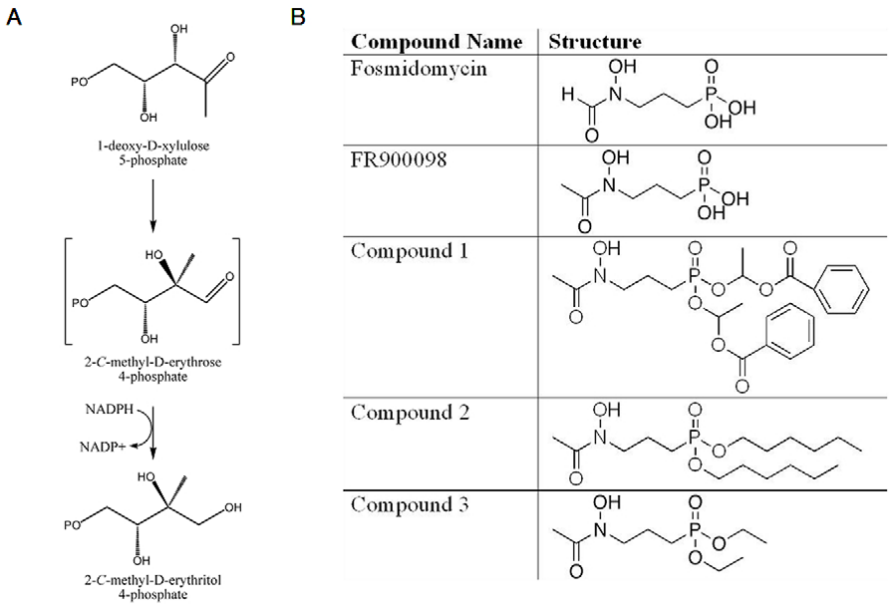
The reaction catalyzed by DXR (MEP synthase) and compounds used in this study. **A.** **MEP synthase pathway**: DXR catalyzes the formation of methylerythritol phosphate (MEP) from 1-deoxy-D-xylulose 5-phosphate (DXP) in an NADPH-dependent mechanism with the formation of the intermediate 2-C-methyl-D-erythrose 4-phosphate. Modified from Jawaid *et al.* [1]. **B: The structures of the compounds used in this study.** Shown are the structures for Fosmidomycin, FR900098, Compound 1, Compound 2 and Compound 3.

The first committed step of the MEP pathway is catalyzed by 1-deoxy-D-xylulose 5-phosphate reductoisomerase (DXR or MEP synthase) [2]. DXR catalyzes the reaction that generates MEP from 1-deoxy-D-xylulose 5-phosphate (DXP) (Figure 1A) [1]. MEP pathway genes have been identified in many biothreat agents, including *Francisella*, *Brucella*, *Bacillus*, *Burkholderia*, and *Yersinia* [1], [3], [4]. DXR has been cloned from many different bacteria, including *Escherichia coli*, *Pseudomonas aeruginosa*, and *Francisella tularensis* [1], [2]. The importance of the MEP pathway to *F. tularensis* is demonstrated by the fact that MEP pathway mutations are lethal [5]. It has also been demonstrated that the DXR gene is essential for *Mycobacterium tuberculosis* [6].

#### 1.1.1 Fosmidomycin and FR900098 are two potent inhibitors of DXR

Fosmidomycin and FR900098 are phosphonate antibiotics naturally produced by *Streptomyces lavendulae* and *S. rubellomurinus*, respectively [7], [8]. The structures of fosmidomycin and FR900098 are related, with FR900098 being the acetyl derivative of fosmidomycin (Figure 1B). Fosmidomycin is currently being used in clinical trials in conjunction with clindamycin to treat malaria [9]. FR900098 is twice as active as fosmidomycin against the malaria parasite *in vitro* and *in vivo* [10]. Two functional groups of these antibiotics are important for their binding efficacy and inhibition of DXR: the hydroxymate moiety that chelates with a divalent metal ion (Mn^2+^, Mg^2+^, or Co^2+^) and a negatively charged phosphonate group [11]. Fosmidomycin and FR900098 are both effective antimalarial agents, but they are limited in their effect due to re-emergence of an active infection after completing treatment [9], and low bioavailability, likely due to low lipophilicity [10]. Fosmidomycin requires the glycerol-3-phosphate transporter (GlpT) to enter cells [12]. Both compounds are well tolerated in mice up to 300 mg/kg and demonstrate antimalarial efficacy following both intraperitoneal and oral administration [13].

We have previously established that fosmidomycin inhibits purified DXR from *F. tularensis* LVS with half maximal activity of 247 nM [1]. This is comparable to its effect against DXR from *M. tuberculosis* (310 nM) [14] and less active against the same enzyme from *E. coli* (35 nM) [2]. Jawaid *et al.* suggest that the difference in concentration required for half-maximal activity may be due to structural differences of the DXR homologs [1].

### 1.2 Lipophilic FR900098 prodrugs

Fosmidomycin and FR900098 are effective against purified DXR from *M. tuberculosis* [6], [14], [15] but have no effect on whole cells of this bacterium because the compounds are too polar to penetrate the thick cell wall [6]. In addition, fosmidomycin requires the GlpT transporter to enter cells, and this is absent in *M. tuberculosis* [14]. This data prompted us to question whether lipophilic analogs of fosmidomycin and FR900098 might better penetrate bacteria with thick cell walls and/or no GlpT transporter. Recently, Ortmann *et al.* generated a series of acyloxyalkyl ester prodrug derivatives of FR900098, including compound 1 (Figure 1B), which demonstrated improved *in vivo* antimalarial activity [10]. These compounds are considered prodrugs of FR900098, and are metabolized to FR900098 in bacteria [10]. We have shown that these analogs have antimicrobial activity against a broad range of bacteria [16] and may also be better at penetrating the cell membranes of eukaryotic cells, which is important for access to intracellular pathogens. For example, both *F. tularensis* and *M. tuberculosis* colonize host cells during the course of infection. We tested the ability of some of these compounds to inhibit *F. novicida* intracellular infection, using both cultured eukaryotic cell lines, and the caterpillar *Galleria mellonella* as an *in vivo* infection model.

### 1.3 *Francisella tularensis*

*F. tularensis* is a highly infectious Gram-negative facultative intracellular bacterium. Inhalation of as few as ten organisms can cause disease in humans [17]. It is of particular interest due to its historical use as a bioweapon, and is on the Centers for Disease Control's list of Category A select agents [18]. *F. tularensis* causes the disease tularemia in mammals, including humans, which can be spread via arthropod vectors, such as ticks [19], or by aerosol. *F. tularensis* can cause a pneumonic disease if it is inhaled, but more commonly causes the ulceroglandular form of the disease that occurs via skin contact. *F. tularensis* can replicate in many different cell types of mammalian hosts (for example, dendritic cells, neutrophils, hepatocytes, and lung epithelial cells), but macrophages appear to be the main target of this bacterium [19], [20]. There are four closely related subspecies of *F. tularensis*, known as *tularensis*, *holarctica*, *mediasiatica*, and *novicida* [19]. *F. novicida* is a model organism of the more virulent *F. tularensis* species [21]. *F. novicida* is attenuated for disease in humans, but can still cause disease in small mammals, such as mice [19]. *F. tularensis* NIH B38 is classified as the type strain for *F. tularensis tularensis*, but is attenuated for virulence [22]–[24], and thus is a good model for *F. tularensis* Schu S4, the fully virulent strain. *F. tularensis* is of concern due to its historical use as a bioweapon in an aerosolized form [18]. Such an event could cause severe pulmonary disease in thousands of individuals and would impose a severe strain and high costs on the health care and public safety systems [20]. Prompt treatment would be important in decreasing the impact of such an attack. The potential of engineered antibiotic resistant strains suggests that new classes of antibiotics with different modes of action from the standard antibiotics, such as ciprofloxacin, should be developed against *F. tularensis*.

### 1.4 Identification of the GlpT homolog in *Francisella*

The uptake of fosmidomycin into many bacteria is an active process dependent on the 12 transmembrane-spanning protein, glycerol-3-phosphate transporter, GlpT [12]. GlpT is a member of the organophosphate:phosphate antiporter family that is part of the major facilitator superfamily (MFS) [12]. *E. coli* mutants defective in the *glpT* gene are resistant to fosmidomycin [12]. *M. tuberculosis* lacks a GlpT homolog, partially accounting for its resistance to fosmidomycin [4], [6]. *Brucella* acquires fosmidomycin sensitivity when it expresses *E. coli* GlpT [4]. We identified a gene in *F. tularensis* (FTT0725c) and in *F. novicida* (FTN_0636) as a potential GlpT homolog (Table 1), and transposon insertion mutants in this locus (Table 2) were tested for their ability to be inhibited by fosmidomycin and analogs.

**Table 1:** GlpT homologs identified in *Francisella spp*.

*Francisella* Species	Locus	Accession Number
*F. tularensis Schu S4*	FTT0725c	YP_169738.1
*F. tularensis LVS*	FTL_1510	YP_514159.1
*F. novicida*	FTN_0636	YP_898283.1
*F. mediasiatica*	FTM_1358	YP_001891977.1
*F. philomiragia*	Fphi_0200	YP_001676919.1

**Table 2:** *F. novicida glpT* transposon insertion mutants used in this study.

Name	Strain	BEI Catalog number
*glpT-1*	tnfn1_pw060323p08q150	NR-5683
*glpT-2*	tnfn1_pw060418p01q161	NR-6558

### 1.5 Hypothesis

The aim of this research is to test the effectiveness of lipophilic FR900098 prodrugs against *F. novicida* as potential platforms for novel antibiotic development. Our hypothesis is that these new compounds will be more effective at crossing biological membranes than FR900098 or fosmidomycin, and thus may be more effective antimicrobial compounds against *Francisella*. Thus, we determined the minimum inhibitory concentration (MIC) and EC_50_ for fosmidomycin, FR900098, and compounds 1–3 against *F. novicida*. In addition, the *in vitro* inhibition of *F. tularensis* LVS DXR by FR900098 was determined in comparison to fosmidomycin [1]. The ability of the lipophilic compounds to cross the *Francisella* membrane was examined using a *glpT* mutant of *F. novicida*, the transporter required for fosmidomycin activity in many bacteria. We then assessed the efficacy of fosmidomycin, FR900098, and compound 1 in treating intracellular *F. novicida* in A549 human alveolar epithelial cells and RAW264.7 mouse macrophages, as well as intracellular *glpT* mutants in A549 cells. The cytotoxicity of the compounds was determined by measuring the LDH released from the infected cells. Finally, *G. mellonella* was used as a model system to test the efficacy of fosmidomycin, FR900098, and compound 1 against *F. novicida* infection *in vivo*. As a lipophilic GlpT-independent DXR inhibitor, compound 1 has the potential to be a broad-spectrum antibiotic, and should be effective against many MEP-dependent organisms [16].

## Results

### 2.1 Susceptibility of *F. novicida* to fosmidomycin, FR900098, and analogs

#### 2.1.1 MICs and EC_50_s of fosmidomycin, FR900098, and lipophilic analogs

The susceptibility of *F. novicida* to the FR900098 analogs was determined in a 96-well plate assay. An initial screening of a panel of compounds demonstrated that compound 1 and compound 2 were the best inhibitors of the analogs tested (data not shown). Compound 3 did not effectively inhibit *F. novicida*, but its structure, along with the others, is included in Figure 1B for comparison.

The MIC values of fosmidomycin, FR900098, compound 1, and compound 2 were determined against *F. novicida* (Table 3). The MIC was best for fosmidomycin at 136 µM. The MIC of FR900098 was 254 µM. The MICs of compound 1 and compound 2 were 202 µM and 1.1 mM, respectively. Thus, compound 1 had approximately 2-fold weaker activity compared with fosmidomycin, but was more active than FR900098.

**Table 3:** Inhibition of bacterial growth by selected compounds.

Compound	Molecular Weight (g/mol)	MIC (µM)	EC_50_ (µM)	EC_50_ 95% Confidence Interval (µM)
**Wild-type ** ***F. novicida***				
Fosmidomycin	183.10 g/mol	136 µM	3.6±0.2 µM	3.1–4.1 µM
FR900098	196.12 g/mol	254 µM	23.2±1.2 µM	21.0–25.5 µM
Compound 1	493.40 g/mol	202 µM	45.2±3.7 µM	38.4–53.2 µM
Compound 2	365.45 g/mol	1094 µM	481±44 µM	401–578 µM
***F. novicida glpT*** ** mutant**				
Fosmidomycin	183.10 g/mol	>1 mM	nd	nd
FR900098	196.12 g/mol	>1 mM	nd	nd
Compound 1	493.40 g/mol	200 µM	nd	nd
Compound 2	365.45 g/mol	>1 mM	nd	nd

MICs and EC_50_s of selected compounds against wild-type and *glpT* mutant *F. novicida* were determined. (nd = not determined).

**Figure 2:**
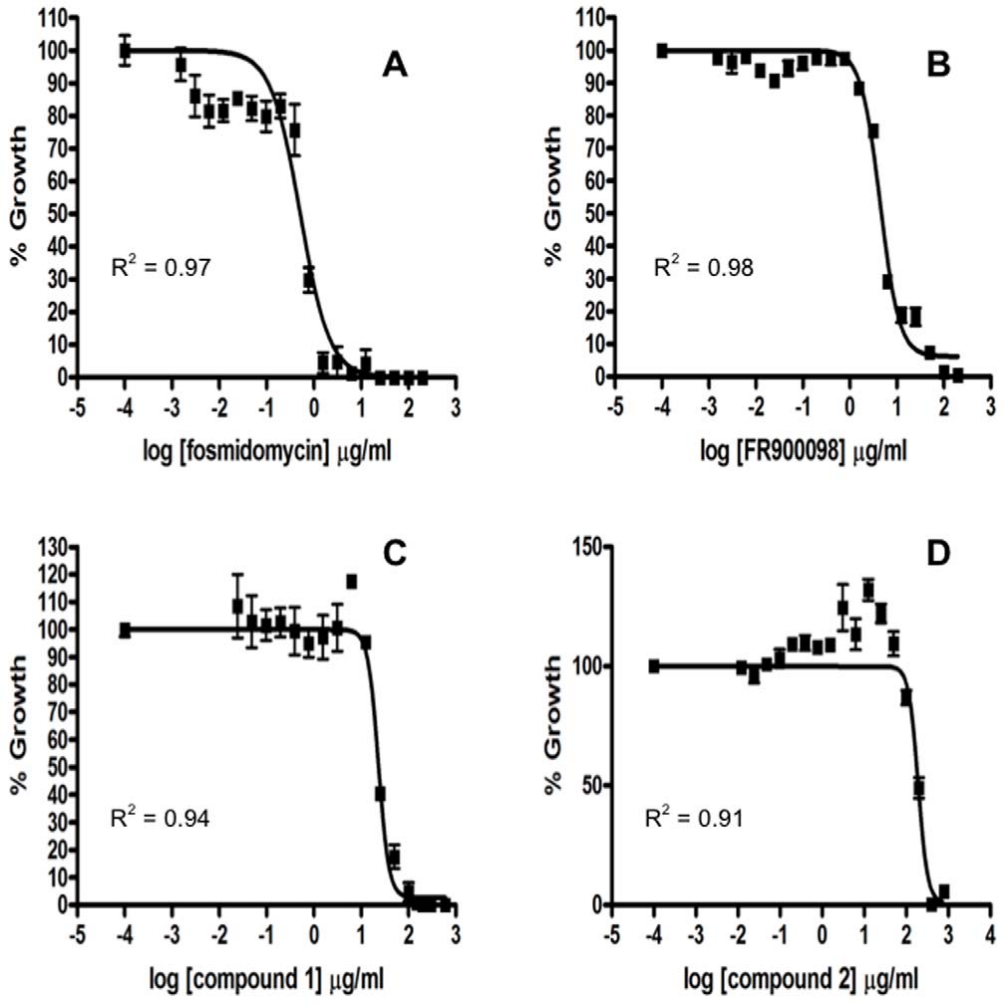
The antimicrobial effect of fosmidomycin, FR900098, compound 1 and compound 2 against *F. novicida*. The EC_50_s (the concentration at which 50% of bacterial growth is inhibited) of fosmidomycin (A), FR900098 (B), compound 1 (C), and compound 2 (D) were determined as previously described. Briefly, serial dilutions of the antibiotic were performed in a 96-well plate format to a defined concentration of bacteria. The percent growth compared to wells with no antibiotic was graphed using GraphPad Prism 4.0. The EC_50_ of fosmidomycin was 3.6±0.2 µM. The EC_50_ of FR900098 was 23.2±1.2 µM. The EC_50_ of compound 1 was 45.2±3.7 µM and the EC_50_ of compound 2 was 481±44 µM.

These compounds were assessed for the number of surviving bacteria at increasing concentration of compound, from which we calculate the EC_50_, a value we use to compare activities of the different compounds. This value was determined for fosmidomycin, FR900098, compound 1, and compound 2. The EC_50_ of fosmidomycin against *F. novicida* was determined to be 3.6 µM (Figure 2A). The EC_50_ of FR900098 was 23.2 µM (Figure 2B). The EC_50_ of compound 1 was 45.2 µM (Figure 2C) and that of compound 2 was 481 µM (Figure 2D). While compound 1 had a better MIC than FR900098, it had a slightly poorer EC_50_.

#### 2.1.2 Inhibition of purified *F. tularensis* LVS DXR by FR900098

The IC_50_ of FR900098 against the DXR of *E. coli* is reported to be 62 nM [25]. The activity of FR900098 was determined by monitoring the enzyme-catalyzed oxidation of NADPH, and using a plot of enzyme fractional activity as a function of inhibitor concentration, as we previously reported, to determine the IC_50_ (Table 4) [1]. The half maximal inhibition of *F. tularensis* LVS DXR by fosmidomycin was previously determined to be 247 nM [1]. FR900098 was found to have an IC_50_ of 230 nM (Figure S1). Since compound 1 is metabolized to FR900098 in bacteria [10], we did not test the activity of compound 1 against DXR *in vitro.* DXR from *Francisella novicida* (FTN_1483), *F. tularensis* LVS (FTL_0534), and *F. tularensis* Schu S4 (FTT1574) share >99% homology (Figure S2). The highlighted differences are not in critical enzymatic residues, thus we conclude that DXR from all Francisella species will have similar sensitivities.

**Table 4:** *In vitro* determination of IC_50_ of selected compounds against *F. tularensis* LVS DXR.

Compound	IC_50_ Inhibition of DXR
Fosmidomycin	247 nM
FR900098	230 nM

#### 2.1.3 Susceptibility of *F. novicida glpT* mutants to fosmidomycin, FR900098, and lipophilic analogs

**Figure 3:**
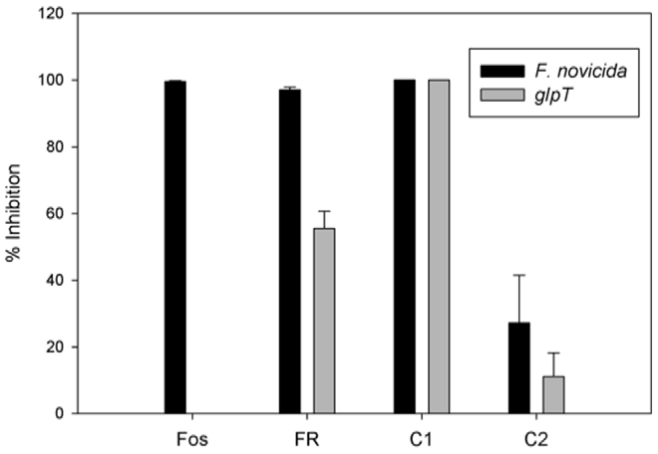
Susceptibility of *F. novicida glpT* mutants to antibiotics. Fosmidomycin, FR900098, compound 1, and compound 2 were tested at concentrations of 200 µg/ml. *F. novicida glpT* mutants were resistant to fosmidomycin and partially resistant to FR900098, but not at all resistant to compound 1. Compound 2 was less effective against the *glpT* mutant than wild-type *F. novicida*. Percent inhibition was calculated by comparing OD_600_ between treated and untreated wells. Fosmidomycin inhibited 99.6±0.2% of wild-type *F. novicida*, but did not inhibit the growth of the *glpT* mutant at all. FR900098 inhibited 97.1±0.8% of *F. novicida*, but only 55±5% of the *glpT* mutant. Compound 1 inhibited 100% of the growth of both wild-type *F. novicida* and the *glpT* mutant. Compound 2 inhibited 27±14% of *F. novicida*, and 11±7% of the *glpT* mutant.

Fosmidomycin requires the glycerol-3-phosphate transporter (GlpT) to enter bacterial cells [6]. Bioinformatic analysis suggests that all *Francisella* species contain GlpT homologs (Table 1). One of the goals of this work was to test compounds that were more efficient at crossing biological membranes independent of the GlpT transporter, presumably by increased penetration of the membrane due to increased lipophilicity. The data for the *glpT-1* mutant strain is shown here (Figure 3), and the data for the *glpT-2* mutant strain was very similar. Even at a concentration of 1 mM, fosmidomycin was not able to inhibit *F. novicida glpT* growth at all (Table 3). At this same concentration, FR900098 was only able to inhibit 50% of *F. novicida glpT* growth. These concentrations inhibited 100% of wild-type *F. novicida* growth. Since inhibition of bacterial growth was reduced against the *glpT* mutant, FR900098 is at least partially dependent on this transporter to enter the bacterial cell. It is possible that FR900098 uses another transporter system, accounting for its 50% inhibition of the *glpT* mutant. Alternatively, FR900098 may be slightly lipophilic and have some ability to cross the membrane independent of GlpT. Compound 2 demonstrated reduced bacteriostatic activity against the *glpT* mutant than wild-type *F. novicida*, suggesting that this analog may also be partially dependent on this transporter to cross the membrane, or a small amount of compound 2 is able to diffuse across the membrane. Both FR900098 and compound 2 are more lipophilic compared with fosmidomycin. In contrast, the MIC of compound 1 was the same for both wild-type *F. novicida* and the *glpT* mutant. This indicates that compound 1 entry into the bacteria is independent of the transporter, and is most likely due to the increased lipophilicity of the acyloxyalkyl ester and its ability to cross the membrane.

### 2.2 Cell line infections and treatments

#### 2.2.1 Infection and treatment of human type II alveolar epithelial (A549) cells

**Figure 4:**
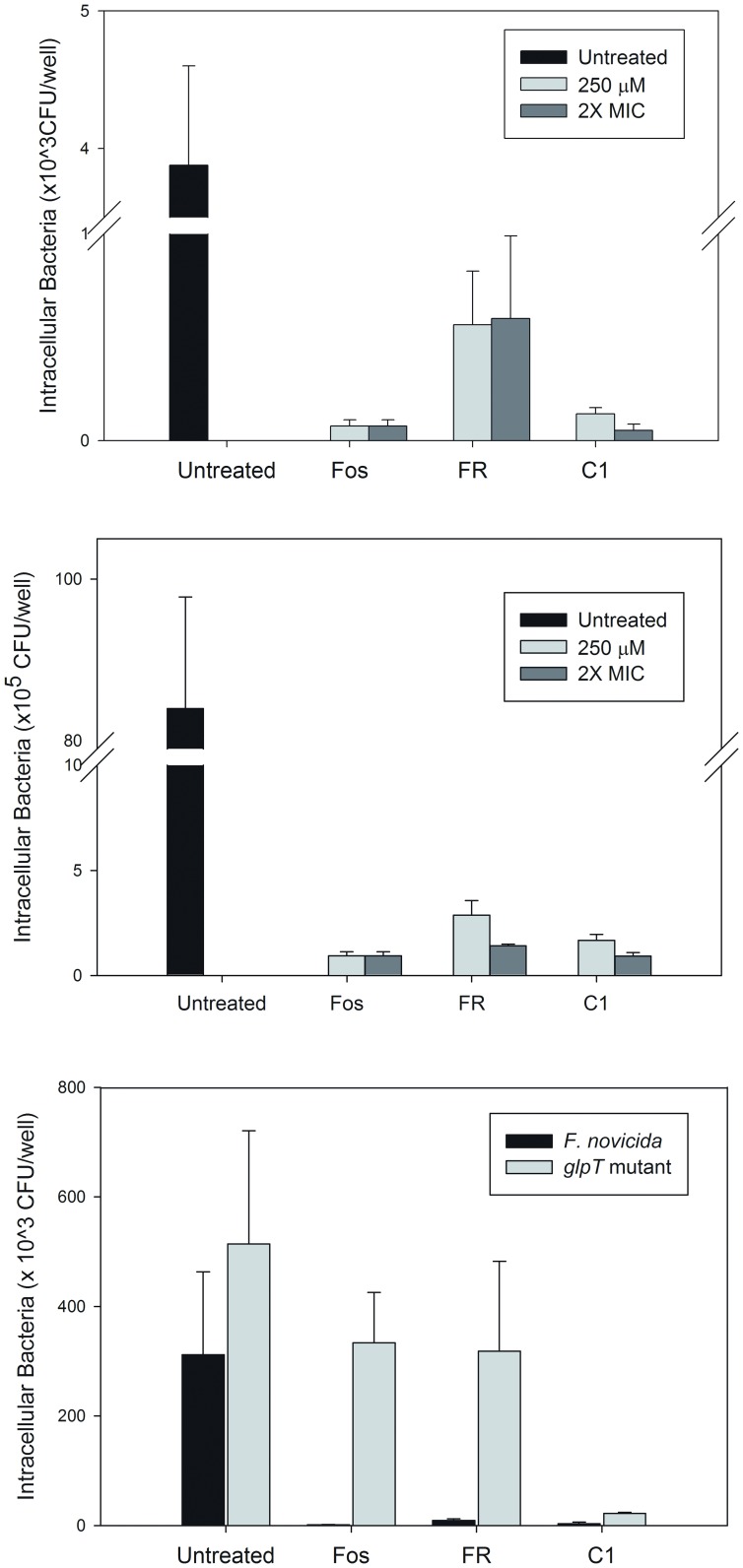
Inhibition of intracellular *F. novicida* replication in two cell lines following treatment with selected compounds. Cell lines were first infected with *F. novicida* at an MOI of 500. The cells were treated with the following concentrations of antibiotics. The cells were lysed after 20 hours of treatment and the intracellular bacteria were enumerated. **A**) **Inhibition of intracellular** ***F. novicida*** **in A549 cells with fosmidomycin (Fos), FR900098 (FR), or compound 1 (C1) for 20 hours.** Each compound was tested at 250 µM and at 2× MIC (2× MIC for fosmidomycin = 250 µM, 2× MIC for FR900098 = 500 µM and 2× MIC for compound 1 = 400 µM). All three compounds significantly inhibited intracellular *F. novicida* growth. At 250 µM, intracellular growth was inhibited 98.0±0.7% by fosmidomycin, 86±6% by FR900098, and 97.0±0.8% by compound 1. At 2× MIC, FR900098 (500 µM) inhibited 85±10% of intracellular growth, while compound 1 inhibited 99.0±0.7% of intracellular growth. **B**) **Inhibition of** ***F. novicida*** **in RAW264.7 cells with fosmidomycin, FR900098, and compound 1 for 20 hours.** Similar results were seen for the RAW264.7 cells as were seen for the A549 cells. **C**) **Inhibition of the** ***F. novicida glpT*** **mutant intracellular replication in A549 cells.** The intracellular-replication inhibition experiment was performed using the *glpT* mutant as previously described for wild-type *F. novicida*. Replication of intracellular *glpT* mutant was not affected by fosmidomycin (250 µM) and FR900098 (500 µM), but was susceptible to compound 1 (400 µM).

When tested at an equal concentration (250 µM), all three antibiotics significantly inhibited the growth of intracellular *F. novicida* (p<0.05) (Figure 4A). Untreated cells contained 3.88±0.72×10^3^ CFU/well of *F. novicida*. At this concentration, fosmidomycin treated cells contained 0.07±0.03×10^3^ CFU/well of *F. novicida* (p<0.0001), representing a 2-log reduction of intracellular bacteria. Cells treated with FR900098 and compound 1 contained 0.56±0.26×10^3^ CFU/well and 0.13±0.03×10^3^ CFU/well, respectively (p<0.0001). In this experiment, fosmidomycin was significantly more effective than both FR900098 and compound 1 (p = 0.005 and 0.006, respectively), likely due to the fact that it was dosed at twice its MIC in the extracellular media. Compound 1 was significantly more effective at inhibiting intracellular *F. novicida* than FR900098 (p = 0.009), likely due to compound 1's extracellular concentration being slightly higher than its MIC (202 µM), while FR900098's extracellular concentration was just at its MIC (254 µM). Although the penetration rate of these compounds across the eukaryotic cell membrane is not precisely known, these results suggest that it was high enough for compound 1 to cross the eukaryotic membrane to achieve intracellular concentrations that approach the MIC.

In a second experiment, each compound was tested at twice the MIC (2× MIC) to encourage maximal diffusion of compound across the eukaryotic cell membrane (Figure 4A). At 2× MIC, compound 1 was as effective as fosmidomycin at inhibiting intracellular *F. novicida*, and was more effective than FR900098. Fosmidomycin at 250 µM was already at 2× MIC, thus its performance did not change. Under these 2× MIC conditions, compound 1 (400 µM) inhibited *F. novicida* intracellular growth to a much greater extent (0.05±0.03×10^3^ CFU/well intracellular bacteria), and was as effective as fosmidomycin. FR900098 (500 µM) did not significantly inhibit more intracellular bacteria (0.59±0.40×10^3^ CFU/well) than at 250 µM, and was the worst performing compound in this assay, suggesting that FR900098 may have some relative inability to penetrate the eukaryotic cell membrane at high efficiency, unlike compound 1. These results demonstrate the antimicrobial activity of compound 1 against *F. novicida* was better than FR900098 and as effective as fosmidomycin.

#### 2.2.2 Infection and treatment of mouse macrophage RAW264.7 cells

The infection and treatment of RAW264.7 cells was carried out as previously described for the A549 cells. Untreated cells contained 84.00±13.8×10^5^ CFU/well of *F. novicida*. Cells treated with 250 µM fosmidomycin contained 0.93±0.20×10^5^ CFU/well (p<0.0001), and those treated with 250 µM FR900098 contained 2.87±0.70×10^5^ CFU/well (p<0.0001). Cells treated with compound 1 had 1.68±0.28×10^5^ CFU/well of intracellular *F. novicida* remaining (p<0.0001). Fosmidomycin was significantly more effective than both FR900098 and compound 1 (p<0.001), and compound 1 was significantly more effective than FR900098 (p<0.01) (Figure 4B).

At 2× the MIC, cells treated with FR900098 (500 µM) had 1.41±0.08×10^5^ CFU/well of intracellular bacteria remaining, which was a significant decrease from 250 µM (p<0.01). Cells treated with 2× MIC of compound 1 (400 µM) contained 0.92±0.18×10^5^ CFU/well. At this concentration, compound 1 was as effective as fosmidomycin (p = 0.94) and significantly more effective than FR900098 (p<0.001) (Figure 4B).

#### 2.2.3 Infection of A549 cells with the *glpT* mutant of *F. novicida* and treatment with fosmidomycin, FR900098, and compound 1

To determine if the *glpT* mutant could be used as an intracellular model for these compounds, A549 cells were infected with *F. novicida* and the *glpT* mutant as previously described. The *glpT* mutant was able to infect cells as efficiently as wild-type (p = 0.10). Fosmidomycin and FR900098 had no effect on the intracellular bacteria (p = 0.12) (Figure 4C). Compound 1 demonstrated a significant effect on the intracellular *glpT* mutant. Untreated cells infected with the *glpT* mutant contained 514.00±206.47×10^3^ CFU/well, while cells treated with compound 1 contained 22.32±2.23×10^3^ CFU/well (p<0.01) (Figure 4C).

#### 2.2.4 LDH assay for determination of cytotoxicity of antibiotics

The release of lactate dehydrogenase (LDH), a stable cytoplasmic protein, can be visualized and quantified in the cellular supernatants when it interacts with a tetrazolium salt (INT) to form a red formazan product. This can be used to measure the percent cytotoxicity of antibiotics against eukaryotic cells. None of the three antibiotics (fosmidomycin, FR900098, compound 1) causes significant cytotoxicity of human lung epithelial A549 cells or mouse macrophage RAW264.7 cells (p>0.05). Treatment did not induce cytotoxicity in either infected or uninfected cells (1.5–4% LDH release, compared with 2.8% LDH release for the control).

### 2.3 *G. mellonella* infection and treatment

**Figure 5:**
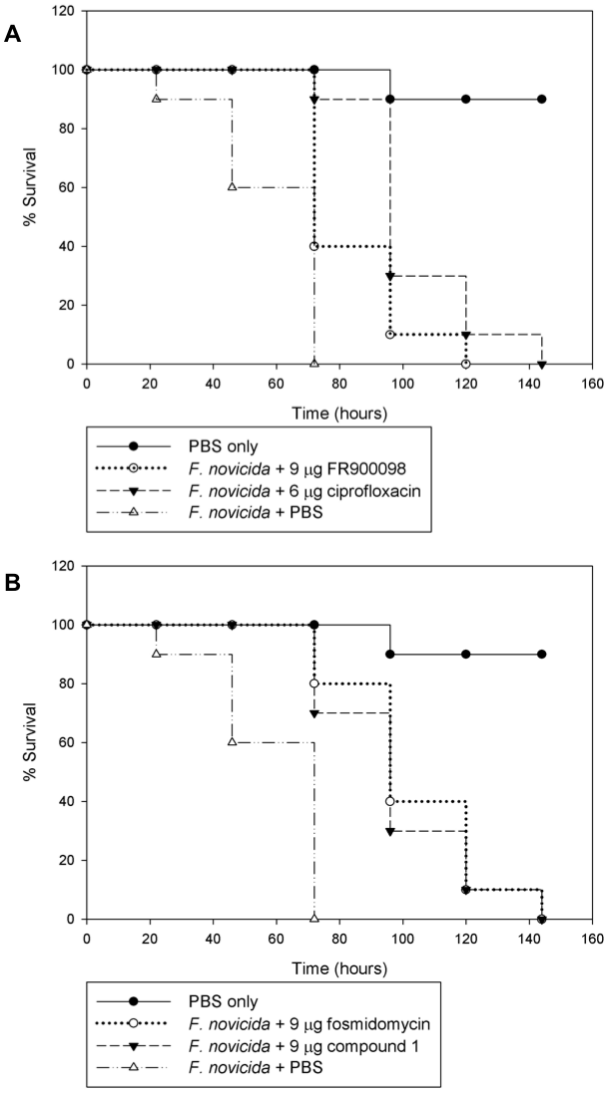
Treatment of *Francisella*-infected wax moth caterpillars with selected compounds. *G. mellonella* were injected with 3×10^4^ CFU of *F. novicida* and treated with 9 µg of antibiotics (or 6 µg of ciprofloxacin). Surviving larvae were counted daily. **A**) **Treatment of** ***G. mellonella*** **with FR900098 and ciprofloxacin.** The mean time to death for untreated caterpillars was 59 hours, and for FR900098 (9 µg)- or ciprofloxacin (6 µg)-treated caterpillars it was 84 hours and 103 hours, respectively. **B**) **Treatment of** ***G. mellonella*** **with fosmidomycin and compound 1.** The mean time to death for caterpillars treated with fosmidomycin or compound 1 (both 9 µg) was 102 hours and 93 hours, respectively.

*G. mellonella* larvae were infected with 3×10^4^ CFU of *F. novicida* by injection into the left proleg. After incubation for 2 hours at 37°C, the larvae were treated with the appropriate antibiotic by injection of 10 µl into the right proleg, as previously described [26], [27]. Fosmidomycin, FR900098, and compound 1 were tested at 30 mg/kg (9 µg per caterpillar), which is the dose that has been used in mice to treat malaria [13]. Ciprofloxacin was used at 20 mg/kg (6 µg per caterpillar) as a positive control [26]. All antibiotics were effective at prolonging the survival of *F. novicida* infected larvae (Figure 5). The mean time to death of untreated larvae was 59 hours. The mean times to death of caterpillars treated with fosmidomycin, FR900098, or compound 1 were 102 hours (p = 0.001), 84 hours (p = 0.009), and 93 hours (p = 0.0006), respectively. The mean time to death for caterpillars treated with ciprofloxacin was 103 hours (p<0.0001). There was no significant difference between the survival of fosmidomycin, compound 1, or ciprofloxacin treated larvae.

## Discussion

The two phosphonate antibiotics, fosmidomycin and FR900098, are both able to effectively inhibit *F. novicida*. Fosmidomycin is able to inhibit *F. novicida* replication *in vitro* with an MIC of 136 µM and an EC_50_ of 3.5 µM. The action of fosmidomycin was confirmed to be absolutely GlpT-dependent in *F. novicida*. FR900098 also inhibited *F. novicida* replication *in vitro* with an MIC of 254 µM and an EC_50_ of 23 µM. However, the action of FR900098 is at least partially GlpT-dependent. Fosfomycin, an antibiotic related to fosmidomycin and FR900098, uses both GlpT and its close homolog, the glucose-6-phosphate transporter (UhpT) in *E. coli* [28]. A BLAST searched revealed that the UhpT transporter is not found in *Francisella*.

The benzoyloxyethyl ester prodrug of FR900098 (compound 1) inhibited *in vitro* replication of *F. novicida* with an MIC of 202 µM and an EC_50_ of 45 µM. Compound 1 was found to be entirely GlpT-independent in its activity, illustrating a significantly improved and novel property of this compound for antimicrobial activity against *Francisella*. This property of compound 1 could be particularly important for bacteria that lack GlpT, such as *M. tuberculosis*. This compound gets metabolized to FR900098 once inside the bacteria.

Compound 2 is similar to compound 1, with a hexyl ester replacing the benzoyloxyethyl ester. Compound 2 was not as effective against *F. novicida in vitro* (MIC ∼1 mM, EC_50_ 481 µM), and its action was partially GlpT-dependent. Thus, *in vitro* compound 2 is less effective than compound 1 and does not demonstrate improved antimicrobial properties against *F. novicida* compared to compound 1. Compound 3 was ineffective against *F. novicida*.

Fosmidomycin is an effective inhibitor of DXR, but may not be an optimal drug candidate because it does not penetrate bacterial membranes without a transporter. Fosmidomycin requires the GlpT transporter to carry it across the membrane to the internal cytoplasm of the bacteria, where it can target DXR. Some bacteria that do not have the GlpT transporter, such as *Brucella*, are relatively unaffected by fosmidomycin [4]. Sangari *et al.* showed that by introducing the GlpT transporter to this organism, the recombinant *B. abortus* became sensitive to fosmidomycin [4]. *M. tuberculosis* (TB) does not have the GlpT transporter, and this may be one of the reasons that fosmidomycin is ineffective against *M. tuberculosis in vitro* [6], in addition to the impenetrability of the mycolic acid layer. Indeed, for *F. novicida*, the GlpT mutants were completely resistant to 1 mM fosmidomycin, indicating that the efficacy of this compound is significantly GlpT dependent. The acetyl analog, FR900098, had better performance in this regard against *F. novicida*, with approximately 50% inhibition against the *glpT* mutant, compared to 100% inhibition of the wild-type *F. novicida*. The lipophilic prodrug of FR900098, compound 1, demonstrated complete GlpT independence, inhibiting 100% of both the wild-type and *glpT* mutant *F. novicida*. Although its efficacy against *Francisella* in the mouse model is not yet known, our results in *G. mellonella* suggest that compound 1 is as effective as FR900098. This finding is similar to those from Ortmann *et al.*, who found that this same compound (compound 1) had antimalarial activity in the mouse model that was similar to, but not better than, FR900098 [10].

A useful result of this study is the development of the *glpT* mutant of *F. novicida* as a model for testing the bacterial-cell penetrating ability of lipophilic fosmidomycin and FR900098 analogs. The *glpT* mutant of *F. novicida* may serve as a model organism to easily screen compounds to identify those analogs with improved membrane penetrating ability (both eukaryotic and prokaryotic membranes). GlpT has not previously been identified as a significant virulence factor of *F. tularensis* using various screens for virulence, intracellular replication, or *in vivo* infection [29]. Other comprehensive studies of genes involved in *Francisella* pathogenesis or intracellular replication have not identified GlpT homologs in any species of *Francisella* as playing a role in these processes [29], [30]. We have also shown that *glpT* mutants can infect and replicate within human lung epithelial A549 cells. Thus, there is no overt role of GlpT in *Francisella* pathogenesis or intracellular replication, supporting the usefulness of this model as a potential screening tool for lipophilic DXR inhibitors.

**Figure 6:**
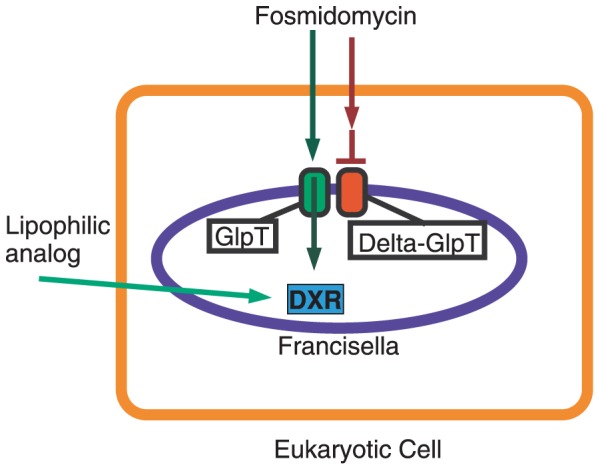
Model for screening method to identify lipophilic, fosmidomycin-derived analogs effective against intracellular pathogens. This is the model for a new screening method for a library of fosmidomycin/FR900098 analogs against host cells infected with intracellular bacteria to identify lipophilic derivatives that can cross both eukaryotic and prokaryotic membranes. In this example, the mammalian cell (orange line) is infected with intracellular bacteria, *F. novicida* (green GlpT) or *F. novicida glpT* mutant (red GlpT) separately. If intracellular bacterial growth of *F. novicida glpT* mutant is inhibited by a compound (inhibition of the DXR enzyme), the fosmidomycin analog is likely able to cross both eukaryotic (orange) and prokaryotic (blue) cell membranes. Such analogs would be good candidates for further testing in other models such as host cells infected with TB. If the analog does not inhibit growth, it may be GlpT-dependent for bacterial cell entry, and thus could not reach the intra-bacterial DXR enzyme in the *glpT* mutant. This would be verified by further testing against wild-type *F. novicida* infected host cells. Thus, by screening eukaryotic cells infected with *F. novicida* glpT mutants, we are able to simultaneously screen for the three critical functional properties of the desired compound (eukaryotic & prokaryotic membrane penetration and GlpT independence).

Our studies with human lung epithelial cells (A549) and mouse macrophages (RAW264.7) demonstrate that fosmidomycin, FR900098 and compound 1 can cross eukaryotic cell membranes efficiently. The LDH assay demonstrates that these compounds are not cytotoxic, suggesting that reduced bacterial counts are due to the antibiotics inhibiting intracellular bacteria, and not due to eukaryotic cell death. Although we did not quantitatively measure the intracellular concentration of these compounds, we can conclude that when concentrations greater than or equal to the MIC are applied extracellularly, the MIC was achieved inside of the eukaryotic cells, as indicated by the complete (fosmidomycin, compound 1) or significant (FR900098) reduction in intracellular bacterial growth. This information suggests that our model using *F. novicida glpT* mutants to screen membrane-penetrating analogs of fosmidomycin can be expanded to be an intracellular infection model, as illustrated in Figure 6.

Our results with the *in vivo G. mellonella* model suggest that fosmidomycin, FR900098, and compound 1 could be effective at treating *Francisella* infections in mammals. The *G. mellonella* infection system has been used to study many human pathogens, including *Francisella* [26], [27]. *G. mellonella* demonstrates a humoral immune response similar to that of mammals, and this response is carried out by hemocytes within the hemolymph of the caterpillar [26]. The host defense response includes phagocytosis, nodulation, and melanization [26], [31]. In the mouse model, 30 mg/kg of fosmidomycin has been used to treat *Plasmodium* infections [13]. This translates to approximately 9 µg per caterpillar. At these concentrations, fosmidomycin, FR900098, and compound 1 were able to prolong the survival of *F. novicida*-infected caterpillars. This suggests that in a mammalian model, these antibiotics could be used to treat *Francisella* infections.

Although the activity of compound 1 against wild-type *F. novicida* is relatively similar to that of fosmidomycin and FR900098, its GlpT-independent activity is of particular interest, especially when considering the problem of antibiotic resistance [32]. For many species of bacteria that cause significant infectious disease, there exists strains that are resistant to commonly used antibiotics [33]. Despite an increase in multiple drug resistant (MDR) pathogens, there has been a dramatic decrease in antibiotic research performed by pharmaceutical companies [34]. No successful discovery of a novel antibacterial agent has occurred since 1987 [35]. Furthermore, resistance to every main class of antibiotic arises within one to ten years of their introduction to clinical use [36], indicating that antibiotic resistance must be planned for.

Antibiotic resistance can occur through many different mechanisms, including the inactivation or modification of drugs, the alteration of the drug target, or efflux pumps that remove drugs from the cytoplasm [37]. Studies suggest that for naturally occurring antibiotics, the rate of resistance is higher than that of synthetic compounds, but horizontal gene transfer can spread resistance for both types of compounds [35], [38]. Thus there is a need for constant antibiotic discovery and improvement on current antibiotics to combat the resistance problem.

The importance of the GlpT-independent activity of compound 1 becomes apparent when considering the possible resistance mechanisms to fosmidomycin and FR900098 [32]. In organisms that utilize the MEP pathway, DXR is an essential enzyme, and mutations are lethal [5], [6] so it is considered a validated drug target. This indicates that antibiotics that target DXR, such as fosmidomycin and FR900098, could potentially be excellent candidates for broad-spectrum use. DXR is an essential enzyme, and while mutations could theoretically occur in the active site that would alter drug activity, loss of this enzyme is unlikely. However, the same cannot be said for the GlpT transporter, as demonstrated by our use of a *glpT* mutant [5], [32]. GlpT is an inorganic phosphate/glycerol-3-phosphate antiporter that depends on the phosphate gradient, so any environmental conditions that affect this gradient could also affect the activity of the transporter [39]. As stated previously, organisms such as *B. abortus* and *M. tuberculosis* use DXR, but lack GlpT [4], [14], thus these compounds may also have utility against these organisms.

A different gene has been identified in *E. coli* that confers resistance to fosmidomycin. It has been labeled *fsr* and is homologous to other drug efflux proteins, such as those that export tetracycline and chloramphenicol [40]. Interestingly, this gene did not confer resistance to fosfomycin, another phosphonate antibiotic dependent on GlpT [40]. A BLAST search revealed that this protein is not present in *F. tularensis*.

Drug targets can be upregulated as a mechanism of resistance. *P. falciparum* that are resistant to fosmidomycin have been shown to upregulate the target enzyme, DXR [41]. This has also been seen as a resistance mechanism of bacteria to fosfomycin, which is in the same class of antibiotic [35]. However, it has recently been shown that fosmidomycin also targets the enzyme following DXR in the MEP pathway, 2-C-methylerythritol-4-phoshpate cytidyltransferase (or IspD) in *E. coli* and *P. falciparum* [42]. This is a promising discovery, as there is a lower frequency of resistance to antibiotics that have multiple targets [35].

Another challenge with both fosmidomycin and FR900098 as therapeutics is their low bioavailability in the serum, probably as a result of their low lipophilicity [10]. The ionization of the phosphonate groups of fosmidomycin and FR900098 at physiological pH is believed to contribute to the low bioavailability of these antibiotics [10], [43]. The plasma concentration of FR900098 was more than twice as high in mice after the administration of an FR900098 prodrug, as opposed to FR900098 on its own [10]. Analogs with higher bioavailability in the serum may be important if bacteria become resistant by upregulating the target enzyme. An antibiotic that has fosmidomycin- or FR900098-like activity, but can freely cross cell walls and membranes, with increased bioavailability, would be an excellent contribution to current therapeutics.

In conclusion, we have shown that the FR900098 prodrug, compound 1, is effective at inhibiting the growth of *F. novicida*, both *in vitro* and *in vivo*. Compound 1 can inhibit intracellular *F. novicida* growth as well as fosmidomycin, and better than FR900098. This prodrug enters the bacterial cell entirely independent of GlpT. Our studies with the *in vivo* model organism, *G. mellonella*, indicate that compound 1 may be effective at clearing mammalian *Francisella* infections. Its effectiveness at treating mice infected with *P. falciparum* further supports this hypothesis [10]. Our collaborators have recently demonstrated the effectiveness of compound 1 against a variety of pathogens [16]. Thus, compound 1 is an effective lipophilic, GlpT-independent prodrug of FR900098 with the potential to be used as a broad-spectrum antibiotic.

## Materials and Methods

### 3.1 Growth of bacteria and tissue culture cells

*F. novicida* (# NR13, BEI Resources, Manassas, VA) was grown in Trypticase Soy Broth with 0.1% cysteine HCl (TSB-C), on TSB-C agar plates, or Chocolate II Agar plates (BD Biosciences) as noted for each assay. *Francisella* on plates was grown in a 37°C incubator with 5% CO_2_. *F. novicida* was grown from bacterial stocks stored in 20% glycerol in a −80°C freezer.

Human epithelial cells A549 (#CCL-185, American Type Culture Collection, Manassas, VA) were grown in Ham's F12 with 10% fetal bovine serum (FBS) in a 37°C incubator with 5% CO_2_. The cells were passed every 3–5 days using trypsin-EDTA and a 1∶3 dilution. RAW 264.7 (#TIB-71, American Type Culture Collection, Manassas, VA) were grown in Roswell Park Memorial Institute (RPMI) media with 10% FBS in a 37°C incubator with 5% CO_2_. The cells were passaged every 3–5 days by scraping at a 1∶3 ratio.

#### 3.1.1 Identification of the GlpT homolog in *Francisella*

The GlpT coding region (GlpT) was identified in the *F. tularensis* SchuS4 (FTT0725c) and *F. novicida* (FTN_0636) genome (accession numbers YP_169738.1 and YP_898283.1) via a BLAST search using the *E. coli* K12 homologous sequence (accession number NP_416743.1) as the query [10]. Transposon insertion mutants in this locus (FTN_0636, Table 1) were obtained through the NIH Biodefense and Emerging Infectious Disease Research Resources Repository, NIAID, NIH: *Francisella tularensis* subsp. *novicida*, “Two-Allele” Transposon Mutant Library. The *F. novicida glpT* mutants were grown as above, with the addition of 10 µg/ml of kanamycin to select for the transposon mutant.

### 3.2 Susceptibility of *F. novicida* to Fosmidomycin, FR900098, and analogs

#### 3.2.1 Synthesis of FR900098 analogs

Compounds 1–3 were made as reported [16]. Synthesis of these compounds was facilitated by referencing strategies in the literature [44]–[48].

#### 3.2.2 Stock solutions of Fosmidomycin, FR900098, and compounds 1–3

All antibiotic stocks were made to have a high starting concentration between 10 and 20 mg/ml. Fosmidomycin (Invitrogen #F-23103) and FR900098 (Sigma-Aldrich # F8307) were obtained as dry powders and were dissolved in water. Compounds 1–3 were dissolved in 100% DMSO to the final concentrations indicated. Gentamicin (Cellgro #61-098-RF), used for cell infection assays, was dissolved in water to a final concentration of 50 mg/ml and was diluted either in Ham's F12 media or RPMI.

#### 3.2.3 Determination of MIC and EC_50_

The minimum inhibitory concentration of an antibiotic compound is the minimum concentration that will completely inhibit all visible growth of bacteria in culture media (bacteriostatic concentration). The EC_50_ is the concentration of compound that will inhibit 50% of bacterial growth. Bacteria were grown in a 96-well flat bottom plate to determine both of these values. To begin the assay, the appropriate amount of compound (or antibiotic) was added to 1×10^5^ CFU/150 µl of *F. novicida* in TSB-C to obtain a final volume of 1 ml and final concentration of compound to 200 µg/ml. The DXR inhibitors were first screened for bacterial growth inhibition by adding 150 µl of the bacteria/antibiotic mixture to a 96-well plate in three separate wells. The plate was incubated at 37°C for 48 hours and read for the MIC assay. Those compounds that inhibited growth were further tested. For the EC_50_ assay, 300 µl of the bacteria/compound mixture were added to three wells. In all other wells there was 1×10^5^ CFU/150 µl of *F. novicida*. 1∶2 serial dilutions were performed in the plate. The plate was wrapped in tin foil to protect it from the light and placed at 37°C with 5% CO_2_ for 24–48 hours. The OD_600_ is read before the plate was placed in the incubator and after 24 or 48 hours.

#### 3.2.4 Data analysis

Data were analyzed using the following equation and GraphPad Prism 4 (GraphPad Software Inc., San Diego, CA).
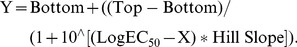
(1)Y corresponds to bacterial mortality (% OD, where zero drug = 100%) at a given antibiotic concentration (µg/ml), with X being the logarithm of that concentration (log µg/ml). In the equation, “Top” and “Bottom” refer to the upper and lower boundaries, and were constrained to values <100% and >0%, respectively. EC_50_ values were determined by fitting the data from the antimicrobial assays to a standard sigmoidal dose-response curve (Equation 1) with a Hill slope of 1. Errors were reported based on the standard deviation from the mean of the log EC_50_ values.

### 3.3 FR900098 IC_50_ determination

The *F. tularensis* LVS DXR was cloned, expressed in *E. coli*, purified, and assayed essentially as described previously [1]. All assays were performed in duplicate, with the 1-deoxy-D-xylulose 5-phosphate (DXP; Echelon Biosciences, Salt Lake City, UT) concentration fixed at the K_M_ (104 µM) and a saturating concentration of NADPH (150 µM). A plot of enzyme specific activity as a function of inhibitor concentration was nonlinear regression fit to a sigmoidal dose-response curve to determine the half-maximal inhibition value (IC_50_) of FR900098. Because fosmidomycin is known to be a slow, tight binding inhibitor of DXR [2], the enzyme was pre-incubated with FR900098 for 10 minutes prior to addition of substrate.

### 3.4 Quantification of intracellular *F. novicida*

#### 3.4.1 Seeding a 96-well plate

A549 and RAW264.7 cells were grown to 75–90% confluence, and then passaged as previously described. Three T75 flasks of cells were combined and the cells were counted using a hemocytometer [49]. They are then spun down (250× g) and resuspended in media for a final concentration of 1×10^5^ cells/100 µl. Cells are added to a 96-well plate so that each well contained 1×10^5^ cells. The plate was incubated overnight (less than 24 hours) at 37°C with 5% CO_2_, allowing the cells to adhere to the plate.

#### 3.4.2 Infection of cells with *F. novicida* and treatment with antibiotics

After overnight growth, A549 or RAW264.7 cells were infected with an overnight culture of *F. novicida* at a multiplicity of infection (MOI) of 500. The MOI is the number of infectious agents divided by the number of cells to be infected. After *F. novicida* is added to the cells, the plate was incubated for 2 hours at 37°C with 5% CO_2_. Extracellular bacteria were then removed by washing the plate 2 times with the appropriate cell culture media. Cells were then incubated for 1 hour with 50 µg/ml of gentamicin to eliminate extracellular bacteria. After this incubation period, cells were again washed twice with media and treated with fosmidomycin, FR900098, or compound 1 at the indicated concentrations. This was the 0 hour time point. The antibiotic solutions also contained 2 µg/ml of gentamicin to prevent extracellular bacterial growth, ensuring that only intracellular bacteria would be counted. The controls (untreated and uninfected) received the 2 µg/ml gentamicin solution with an appropriate amount of DMSO, to account for any effect of the DMSO in the compound 1 solution.

#### 3.4.3 Quantification of intracellular *F. novicida*

Intracellular *F. novicida* was quantified at 0 and 20 hours. For the 0 hour time point the concentration of intracellular *F. novicida* was determined immediately after the addition of the antibiotics. For 20 hour time point, the cells were incubated for an additional 20 hours at 37°C, 5% CO2. The media was aspirated and the cells were washed twice with 1× PBS. Cells were lysed to release the intracellular bacteria by the addition of 100 µl of sterile distilled water and serially diluted in 1× PBS. Dilutions were plated on TSB-C agar plates and incubated at 37°C with 5% CO_2_ for 24 hours. Colonies were counted to determine the CFU/well of intracellular bacteria that remained following each treatment.

#### 3.4.4 LDH assay for determination of cytotoxicity of antibiotics

The cytotoxicity of antibiotics against eukaryotic cells can be determined by measuring the release of cytoplasmic proteins, which is a sign of apoptosis. Lactate dehydrogenase (LDH) is a stable cytoplasmic protein that is released when the cell is lysed. LDH can be visualized and quantified in the cellular supernatants when it interacts with a tetrazolium salt to form a red formazan product, which has absorbance at 490 nm. The CytoTox-96 ® Non-radioactive Cytotoxicity Assay kit (Promega) was used to quantitatively measure lactate dehydrogenase (LDH) release at 22 hours, following the manufacturer's instructions. Absorbance values were recorded at 490 nm by spectrophotometer (μQuant, BioTek). Background values were subtracted from sample readings. The percent cell death was determined using the formula in **Equation 2**.
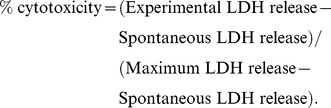
(2)


### 3.5 *Galleria mellonella* infection and treatment

*G. mellonella* (wax moth caterpillars) larvae were used to test the *in vivo* effect of fosmidomycin, FR900098, and compound 1 against *F. novicida* infection. *G. mellonella* larvae were used in their final larval stage and stored at room temperature in the dark. Ten caterpillars of 0.30–0.35 g were used in each group. The following control groups were used for each experiment: caterpillars that received no injection, caterpillars injected with 1× PBS to determine any mortality associated with the trauma of injection, caterpillars injected with PBS and antibiotics to determine any mortality caused by antibiotics alone, and caterpillars injected with bacteria and “treated” with 1× PBS. All injected caterpillars received two injections. A 1 cc tuberculin syringe was used for all injections. The syringe was used to inject 10 µl of 3×10^6^ CFU/ml of *F. novicida* into the hemocoel via the left proleg of all caterpillars to be infected. Control caterpillars were injected with 10 µl of 1× PBS in the left proleg. Caterpillars were then incubated at 37°C for 2 hours. After the incubation period, the next round of injections was performed using the right proleg. Control caterpillars received either 10 µl of 1× PBS or 10 µl of antibiotic. Treated caterpillars received 10 µl of antibiotic. Fosmidomycin, FR900098, and compound 1 were tested at the concentrations indicated in the results section. Live caterpillars were counted daily. The mean time to death was determined for each group.

### 3.6 Statistical analysis

Two tailed T-tests were performed to determine statistical significance of bacterial infection and LDH release data, assuming unequal variance in the different populations. For *in vivo* testing in *G. mellonella*, Kaplan-Meier survival analysis was used (http://www.medcalc.be/).

## Supporting Information

Figure S1 **IC_50_ of FR900098 against DXR.** Nonlinear regression fitting the sigmoidal dose-response curve resulted in an IC_50_ of 230 nM, slightly more potent than fosmidomycin (EC_50_ = 247 nM) [1]. All assays were performed in duplicate. The R^2^ value is indicated.
(TIFF)

Figure S2 **Amino acid alignment of DXR from** ***Francisella*** **species.** The DXR from *Francisella novicida* (FTN_1483), *F. tularensis* LVS (FTL_0534), and *F. tularensis* Schu S4 (FTT1574) share >99% homology. The highlighted differences are not in critical enzymatic residues.
(TIFF)
