# Supplementary figures and images for: Lipophilic Prodrugs of FR900098 Are Antimicrobial against Francisella novicida In Vivo and In Vitro and Show GlpT Independent Efficacy
Source: PLoS One. 2012 Oct 15;7(10):e38167. doi: 10.1371/journal.pone.0038167 (PMC3471904; doi:10.1371/journal.pone.0038167)

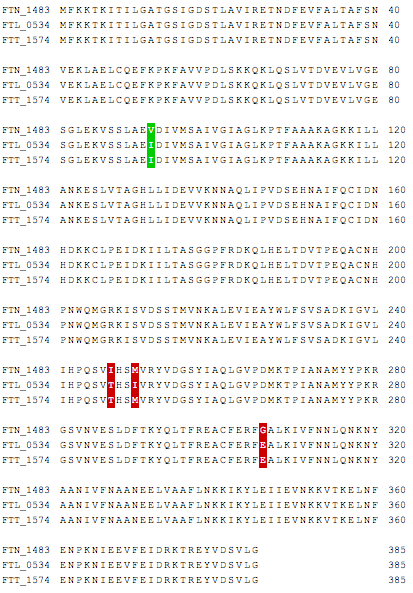

Supplement: Figure S2 — Amino acid alignment of DXR from Francisella species. The DXR from Francisella novicida (FTN_1483), F. tularensis LVS (FTL_0534), and F. tularensis Schu S4 (FTT1574) share >99% homology. The highlighted differences are not in critical enzymatic residues. (TIFF) [file pone.0038167.s002.tif]
